# 

**Published:** 1842-12-17

**Authors:** 


### CONTENTS OF No. 51.

Dr. Folt's case of Audition restored by the Removal of a tumor from Hyo-Thyroid Region, - - - - - - - - 801

ANALECTA: On the starched Bandage in Fractures, - - - - 802
Extract from Dr. O'Beirne's Observations on the Nature and Treatment of Dropsy, - - - - - - - 807
Mr. Coulson on Paralysis of the Bladder, - - - - 810
Treatment of Hæmaturia, - - - - - - 812
Typhus in Old Age, - - - - - - - - 812

BIBLIOGRAPHICAL NOTICE: On the Usefulness of the Medical Profession, beyond the limits of the Profession. A Lecture Introductory to the Course of Practice of Medicine, in Jefferson Medical College of Philadelphia. Delivered November 4, 1842. By J. K. Mitchell, A.M. M.D. - - - 813

